# Decreased Expression of CoREST1 and CoREST2 Together with LSD1 and HDAC1/2 during Neuronal Differentiation

**DOI:** 10.1371/journal.pone.0131760

**Published:** 2015-06-25

**Authors:** Julián Esteban Sáez, Andrea Verónica Gómez, Álvaro Patricio Barrios, Guillermo Eduardo Parada, Leopoldo Galdames, Marcela González, María Estela Andrés

**Affiliations:** Department of Cellular and Molecular Biology, Faculty of Biological Sciences, Pontificia Universidad Católica de Chile, Santiago, Chile; Indian Institute of Toxicology Reserach, INDIA

## Abstract

CoREST (CoREST1, *rcor1*) transcriptional corepressor together with the histone demethylase LSD1 (KDM1A) and the histone deacetylases HDAC1/2 form LSD1-CoREST-HDAC (LCH) transcriptional complexes to regulate gene expression. CoREST1 belong to a family that also comprises CoREST2 (*rcor2*) and CoREST3 (*rcor3*). CoREST1 represses the expression of neuronal genes during neuronal differentiation. However, the role of paralogs CoREST2 and CoREST3 in this process is just starting to emerge. Here, we report the expression of all CoRESTs and partners LSD1 and HDAC1/2 in two models of neuronal differentiation: Nerve-Growth-Factor (NGF)-induced neuronal phenotype of PC12 cells, and *in vitro* maturation of embryonic rat cortical neurons. In both models, a concomitant and gradual decrease of LSD1, HDAC1, HDAC2, CoREST1, and CoREST2, but not CoREST3 was observed. As required by the study, full-length rat *rcor1* gene was identified using *in silico* analysis of available rat genome. The work was also complemented by the analysis of rat RNA-seq databases. The analysis showed that all CoRESTs, including the identified four splicing variants of rat CoREST3, display a wide expression in adult tissues. Moreover, the analysis of RNA-seq databases showed that CoREST2 displays a higher expression than CoREST1 and CoREST3 in the mature brain. Immunofluorescent assays and immunoblots of adult rat brain showed that all CoRESTs are present in both glia and neurons. Regarding functional partnership, CoREST2 and CoREST3 interact with all LSD1 splicing variants. In conclusion, neuronal differentiation is accompanied by decreased expression of all core components of LCH complexes, but not CoREST3. The combination of the differential transcriptional repressor capacity of LCH complexes and variable protein levels of its different components should result in a finely tuned gene expression during neuronal differentiation and in the adult brain.

## Introduction

The corepressor CoREST, the histone demethylase LSD1 (also known as KDM1A), and the histone deacetylases HDAC1/2 are the core components of the LSD1-CoREST-HDAC (LCH) transcriptional repressor complexes [1,2]. Recent data has shown a wide diversification of the LCH complexes by including the LSD1 splicing variants and a family of CoREST genes. LSD1 exists in four splicing variants in mammalian genomes, LSD1, LSD1-2a, and the exclusive neuronal LSD1 variants containing a loop of four amino acids encoded by exon 8a (LSD1-8a and LSD1-2a/8a) [3]. On the other hand, in mammalian genomes, three independent genes, *rcor1*, *rcor2*, and *rcor3* encode CoREST1 (previously called CoREST), CoREST2 and CoREST3, respectively. Moreover, four splicing variants have been described for human CoREST3.

Crystallographic [4,5] and biochemical evidences [6,7] indicate that the core components of the LCH complexes constitute multimeric entities to epigenetically modify H3 histone tail. However, the transcriptional output will depend on the specific components constituting the complex. We have recently shown that LCH complexes formed with CoREST2 exhibits less histone deacetylase activity and CoREST3 containing complexes show reduced LSD1 catalytic efficiency [8]. Accordingly, CoREST2 and CoREST3 containing complexes display lower transcriptional repressive capacity compared with CoREST1 containing complexes. In addition, it was shown that the shortest CoREST3 splicing variant display a dominant-negative effect compared to other CoRESTs containing complexes during blood cells differentiation [9].

During last years, accumulated evidence shows the importance of CoREST1, LSD1 and HDAC1/2 in neuronal differentiation. CoREST1 is a corepressor of the RE-1 silencing transcription factor/Neural Restrictive Silencing Factor (REST/NRSF), essential to repress the expression of neuronal genes in non-neuronal cells [10,11]. CoREST1 bound to REST/NRSF is also important to maintain repressed neuronal genes in neuronal stem cells [12]. Independent of REST/NRSF, CoREST1 regulates the expression of a specific set of genes in mature neurons [12,13]. In addition, CoREST1 plays a significant role in cortical neuronal migration, process that also depends on LSD1 [14]. Few reports have addressed the role of CoREST2 and CoREST3 in neuronal differentiation. Studies in *Xenopus* suggest that CoREST2 may play a redundant role to CoREST1 during neuronal differentiation. Indeed, X*enopus* CoREST2 is able to interact with REST/NRSF and to regulate neuronal differentiation [15]. Interestingly, it has been also shown that CoREST2 is essential to maintain pluripotency and proliferation capacity in embryonic stem cells [16].

LSD1 also plays a key role during early events of development and maintenance of the pluripotency [15] and for terminal differentiation of several cell phenotypes [16,17]. Neuronal LSD1 variants contribute to the terminal differentiation of hippocampal neurons *in vitro* [3]. Moreover, the phosphorylation of the threonine 369 found in the 8a-encoded four amino acid loop enhances the morphogenic function of the neuronal LSD1. This post-translational modification prevents the interaction with CoREST1 and HDACs, transforming neuronal LSD1 in a variant with a dominant-negative function [18].

HDAC1 and HDAC2 are also developmentally regulated and play a major role during nervous system maturation. The deletion of both HDAC1 and HDAC2 in developing neurons results in severe brain abnormalities and early postnatal lethality [17]. Recently, it was shown that HDAC2, but not HDAC1, plays an essential role controlling neural progenitors destination during brain development [18]. HDAC1 is expressed at higher levels than HDAC2 in cerebellar progenitors; relation that is reverted in post-mitotic neurons [19].

All these data suggest that different LCH complexes participate in cell differentiation process and in differentiated cells. Here we describe the pattern of expression of the core components that constitute LCH complexes during neuronal differentiation in the NGF-induced neuronal phenotype of PC12 cells and *in vitro* maturation of rat cortical neurons. Using these models, we show that CoREST1 and CoREST2 decrease together with LSD1 and HDAC1/2 while CoREST3 remains unchanged during neuronal maturation. Furthermore, we complement these data analyzing available RNA-seq databases showing the wide and distinct expression of each CoREST in adult rat tissues.

## Materials and Methods

### Animals

E14.5 and E18.5 embryos and adult Sprague–Dawley rats (250–300 grams) were used for the study (Animal Care Facility of the Faculty of Biological Sciences, Pontificia Universidad Catolica de Chile). Day 0.5 of pregnancy was established by the presence of spermatozoa in the vaginal smear. Pregnant rats were decapitated with a guillotine and embryos obtained by cesarean section. Embryos were decapitated with scissors and cortices or whole brain dissected out for immediately processing for cell culture or total RNA extraction. Adult male rats (250–300 gr) were decapitated, and brains immediately processed for total RNA or protein extraction. Every effort was made to reduce the chance of pain or suffering. The procedures were approved by the Bioethical Committee of the Faculty of Biological Sciences of the Pontificia Universidad Católica de Chile. The procedures were performed in strict accordance with the guidelines and policies established in the Chilean Institutional Bioethical guide (Comisión Nacional de Investigación Científica y Tecnológica, Conicyt).

### Cell culture conditions and primary culture of cortical neurons

PC12 cells (ATCC) were cultured in Dulbecco’s modified Eagle’s medium (DMEM) supplemented with 10% horse serum, 5% fetal bovine serum (FBS), 1% penicillin/streptomycin and maintained at 37°C and 10% CO2. PC12 cells were plated at 5x10^3^ cells/cm^2^ density. Twenty-four hours later, the medium was replaced with DMEM supplemented with 2% horse serum plus 50 ng/ml of NGF (Alomone Labs, Ltd) to induce neuronal differentiation. Cells were maintained for seven days, changing the media with fresh NGF every two days. Undifferentiated PC12 cells were cultured under the same low-serum conditions but without NGF. HEK293T cells (ATCC) were cultured in DMEM with 10% FBS, 1% penicillin/streptomycin and maintained at 37°C and 5% CO2.

Primary cultures of cortical neurons were obtained by cortices dissection and tissue dissociation in HANK´s medium with papain and DNase. After 20 min incubation at 37°C, the medium was replaced by adhesion solution (MEM with 10% FBS, 2 mM glutamine and 30 mM glucose) and cells pipetted up and down with a Pasteur pipette. Cortical cells were plated on poly-L-lysine-coated wells and maintained in Neurobasal medium supplemented with B27, 100 U/ml penicillin and 100 μg/ml streptomycin for 2–4 or 6 days *in vitro* (DIV) before any experimental manipulation. Two μM Cytosine-Arabinoside (AraC) was added to inhibit glial proliferation, on the second day of culture, and removed by changing the medium 24 h later. Immunofluorescence assays were carried out on cultures either treated or untreated with AraC in order to determine the proportion of glial cells present in the cortical cultures. Unless otherwise indicated, all cell culture reagents were acquired from Invitrogen Corporation.

### Protein co-immunoprecipation assays and plasmid transfection

HEK293T cells (3.5x10^6^ cells) were co-transfected with 2.5 μg myc-CoREST2 or myc-CoREST3 and 2.5 μg of each HA-LSD1 isoform (kindly donated by Dr. Elena Battaglioli) using OptiMEM (Life Technologies) and Lipofectamine 2000 reagent (Invitrogen Corporation), and harvested 48 h post-transfection. Co-immunoprecipitation assays and immunoblots were carried out as described [8,20].

### Antibodies

anti-CoREST1 antibodies K72/8 and 612146 were obtained from Neuromab and BD Transduction Laboratories, respectively. Anti-HDAC1 (10E2), anti-HDAC2 (3F3), anti-CoREST3 (ab76921), anti-LSD1 (ab17721) antibodies were obtained from Abcam. Anti-LSD1 (2139) and anti-GAPDH were obtained from Millipore. Anti-CoREST2 (HPA021638) and β-actin (A2228) antibodies were obtained from Sigma. Histone H3 (sc-10809), anti-HA (sc-805) and anti-Myc (9E10) antibodies were obtained from Santa Cruz Biotechnology. Anti-HA (MMS-101P) antibody was obtained from Covance.

### Protein extraction and immunoblotting

PC12 and embryonic cortical neurons were washed three times and scrapped in ice-cold phosphate-buffered saline (PBS). Mixed glia (astrocyte and microglia) cultures, obtained as described [21], were generously donated by Ms. Francisca Cornejo (Dr. Rommy von Bernhardi laboratory, P. Universidad Católica de Chile). Total protein extracts were obtained by lysing the cells with 1ml syringe in Triton X-100 lysis buffer (50mM Tris-HCl pH 7.5, 1% Triton X-100, 150mM NaCl, 1mM PMSF plus protease inhibitors), centrifuged at 14000 rpm for 20 min at 4°C and supernatants saved for further analysis. Protein samples were resolved on SDS-PAGE and visualized by immunoblot using specific antibodies. Quantification was performed with ImageJ software using GAPDH, H3 or β-actin as loading control. The nuclear fraction from PC12 cells was obtained as described previously [22] with one modification. After washing the nuclear fraction, the pellet was resuspended in Triton X-100 lysis buffer, as described above.

### Immunofluorescence assays

Immunofluorescence assays were performed as we have described [23,24]. Brain slices were incubated with anti-CoREST1 (612146 from BD Transduction Laboratories) during 48 h at 4°C and then anti-CoREST2 (HPA021638 from Sigma) antibody was added followed by 12 h of incubation. Subsequently, slices were mounted on glass slides and incubated with secondary anti-rabbit IgG coupled to Alexa 488 and anti-goat IgG coupled to Alexa 594. Finally, analysis and photomicrography were carried out with confocal microscopy (Olympus FV-1000). For immunofluorescence assay in primary culture of cortical neurons, anti-ß-III Tubulin (sc-816, Santa Cruz Biotechnology) and anti-GFAP (Dako z0334) were used.

### Semiquantitative RT-PCR

Total RNA was extracted from cells or tissue using TRIzol reagent (Invitrogen Corporation) following manufacturer’s instructions; 2μg of RNA were subjected to reverse transcription using MMLV-RT (Fermentas International Inc) and amplified using the following primers:

*rcor2*-forward: 5’-GCAGTTGAGCTTGTAAACCC-3’

*rcor2-*reverse: 5’-CAACATTCTTCCCACAAGGC-3’

*rcor3*-(A)-forward: 5’-ATGACCCAAAGAAAGAAGCC-3’

*rcor3*-(A)-reverse: 5’-GTTAGATACATGCCCTTAGG-3’

*rcor3*-(B)-forward: 5’-GCCAACAGACACAATCAAGG-3’

*rcor3*-(B)-reverse: 5’-AGACTCTGGTACTCTCTCC-3’

*rpl19-*forward: 5’-ACCTGGATGCGAAGGATGAG-3’

*rpl19*-reverse 5’-ACCTTCAGGTACAGGCTGTG-3’

Quantification was performed with ImageJ software using *rpl19* as reference gene.

### Bioinformatic analyzes

We used public available RNA-seq data from eight tissues extracted from 3 different rats [25]. Rat RNA tissue samples were sequenced by an Illumina HiSeq 2000 platform; two rats were sequenced with a 35–50 bp paired-end protocol and one rat was sequenced with a 75–80 bp paired-end protocol. We trimmed the low-quality sequences from the 75–80 bp RNA-seq reads with sickle (available at https://github.com/najoshi/sickle). We used Tophat2 [26] to align the reads to the rat reference genome (rn5). The read alignments located at the RCORs loci were assembled with Cufflinks [27]. To visualize and analyze assembled transcripts we used the UCSC Genome Browser database [28]. To annotate protein domains at the assembled transcripts, we integrate the rat, mouse and human protein information available at UniProt [29].

### Statistical analysis

Non-parametric Mann-Whitney U test or One-way analysis of variance (ANOVA) for 2 groups, and Two-way ANOVA followed by Bonferroni post hoc test for multiple groups comparison were used to determine statistical significance of the differences, using Prism software.

## Results

### Identification of rat rcor1 gene and the splicing variants of CoREST3

**Fig 1:**
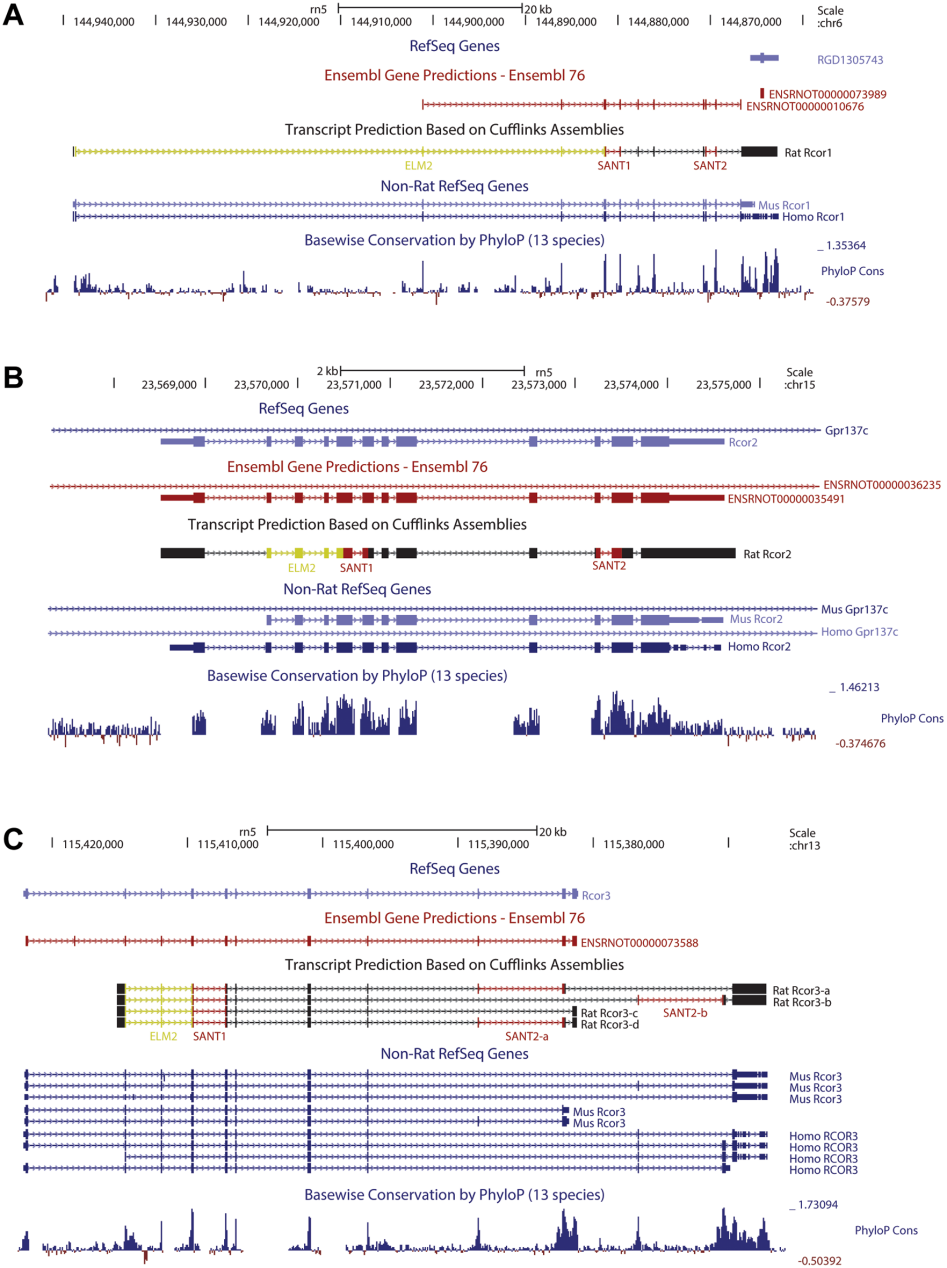
Annotation of rat *rcor1-3* transcripts. UCSC Genome Browser images show transcript annotation data at *rcor1* (A), *rcor2* (B) and *rcor3* (C) loci. Gene model tracks of RefSeq and Ensembl are shown in blue and red, respectively. Assembled transcripts by Cufflinks are shown in black. Predicted ELM2 and SANT1/SANT2 domains are highlighted in yellow and red, respectively. Together, the alignments of RefSeq transcripts annotation from human and mouse with the PhyloP genomic conservation scores give the evolutionary information to support the existence of the new identified exons.

Rat rcor1-3 transcripts expression was analyzed using public available RNA-seq data from eight different tissues [25]. Using Cufflinks [27] we assembled the RNA-seq read alignments at the rcor1-3 loci. The assembled transcripts showed a strong evolutionary correspondence with the rcor1-3 transcripts of mouse and human models (Fig 1A). At rcor1 locus, whereas RefSeq [30] track shows a single unspliced transcript, Ensembl [31] track shows a ten-exon transcript that is coincident with mouse and human rcor1 transcript model. Our transcript assembly of rcor1 allowed identifying two additional exons that were not annotated at Ensembl gene model. These additional exons codify for the ELM2 domain of rat CoREST1 (Fig 1A). At rcor2 locus, the assembled transcript is coincident with the rcor2 gene models of RefSeq and Ensembl (Fig 1B). Finally, at the rcor3 locus, we assembled three rcor3 isoforms that are not annotated in RefSeq or Ensembl. Interestingly, one of the novel isoforms (rcor3-b) contains three unannotated exons, and two of them codify for an additional SANT2 (SANT2-b) domain that is only included in this isoform. None of the new assembled rcor3 transcripts contains both SANT2 domains, suggesting that the inclusion of the pair of exons that codify for the SANT2 domains are mutually exclusive (Fig 1C). Duplication of SANT2 domains at rcor3 locus were not detected in human or mouse (S1 Fig).

### *In silico* analysis of CoRESTs expression in adult rat tissues

**Fig 2:**
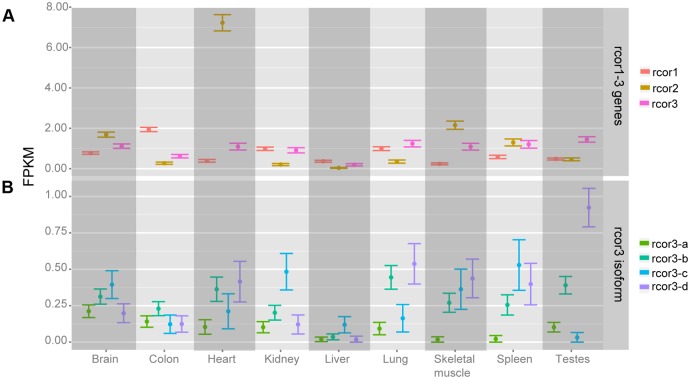
*rcor1-3* transcripts expression profile across rat tissues. The panel shows the expression levels (measured in FPKM) of *rcor1-3* genes (A) and *rcor3* isoforms (B) in the different rat tissues analyzed.

To assess rcor genes expression patterns, we performed an in silico analysis of available databases from adult rats. As shown in Fig 2, all rcors including the 4 splicing variants of rcor3 are expressed in all analyzed tissues. The expression of rcor2 is remarkably higher in the heart compared to all other tissues, being ten times higher than rcor1 and rcor3. In the adult rat brain, all rcors are expressed, and rcor2 displays a higher expression than its paralogues (Fig 2).

### PC12 neuronal differentiation down-regulates LCH components

**Fig 3:**
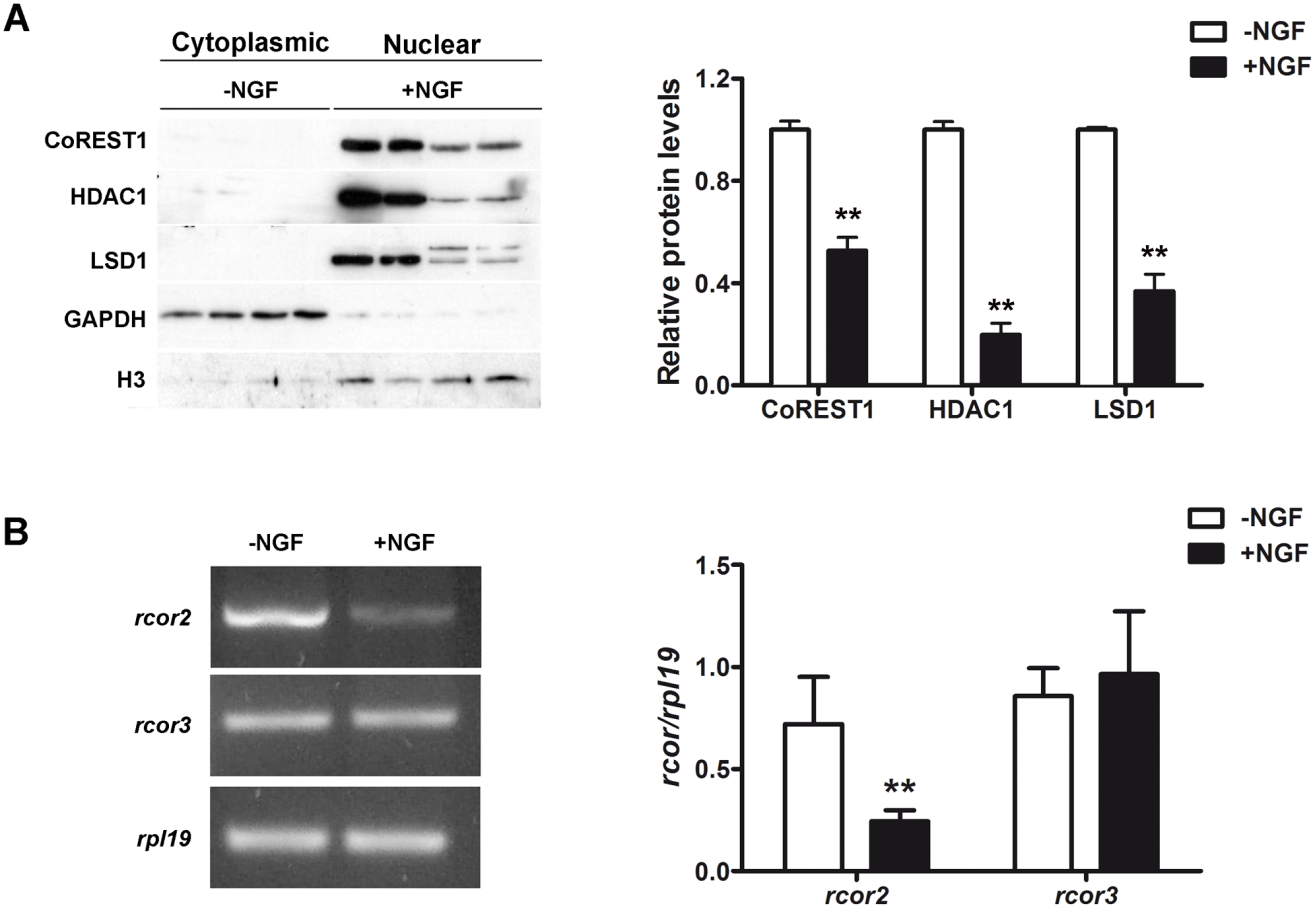
CoREST1 and CoREST2, but not CoREST3 are down-regulated during NGF-dependent neuronal differentiation of PC12 cells. (A) Left: representative immunoblots of CoREST1, HDAC1 and LSD1 in equivalent protein amounts of cytosolic and nuclei fractions of control (-NGF) and NGF treated (50 mg/ml during 7 days) PC12 cells. Histone H3 was used as loading control for nuclear fraction and GAPDH for cytoplasmic fraction. Right: graph of relative protein levels of CoREST1, HDAC1 and LSD1 respect to histone H3 levels. Values correspond to the mean ± SEM of at least 3 independent experiments. **p<0.01. Statistical analysis performed by Mann-Whitney test. (B) Semiquantitative RT-PCR was performed for rat *rcor2* and *rcor3* genes, *rpl19* gene was used as reference gene. Left: representative PCR reaction. Right: Quantification of *rcor2* and *rcor3* mRNA expression levels in control (-NGF) and NGF treated PC12 cells. Values correspond to the mean ± SEM. **p< 0.01. Statistical analysis performed by Mann-Whitney test.

Cell differentiation processes require morphological and functional changes involving gene reprogramming. Thus, the modulation of transcriptional complexes activity is a major regulatory mechanism during cell differentiation. We evaluated LCH components expression in the PC12 cells neuronal differentiation model. NGF treatment (50 mg/ml) of PC12 cells during 7 days induced neuronal-like phenotype acquisition [32] characterized by dendrite growth, a network appearance of cultured cells, cell proliferation arrest and increased expression of GAP43, specific neuronal marker (S2 Fig). After 7 days of incubation with NGF, a significant decrease of CoREST1 and LSD1 proteins plus a remarkable 80% decrease of HDAC1 were observed in the neuron-like differentiated PC12 cells (Fig 3A). Interestingly, a slower migrating band recognized by the anti-LSD1 antibody appears in the nuclear fraction of NGF-treated PC12 cells, which match the molecular weight of 2a-exon containing LSD1 variant [3]. As expected, CoREST1, HDAC1, and LSD1 presented a predominant nuclear localization that was not modified after the NGF treatment (Fig 3A).

To analyze whether *rcor2* and *rcor3* expression are subjected to regulation during PC12 neuronal differentiation, we measured the expression of *rcor2* and *rcor3* (all predicted splicing variants, see S4 Fig for amplicons localization) by RT-PCR. Similar to CoREST1, *rcor2* is abundantly expressed in undifferentiated PC12 cells and decreases significantly in NGF-treated PC12 cells (Fig 3B). Interestingly, *rcor3* expression does not change after NGF-induced neuronal differentiation of PC12 cells (Fig 3B).

### *In vitro* maturation of rat cortical neurons decreases the expression of specific components of LCH complexes

**Fig 4:**
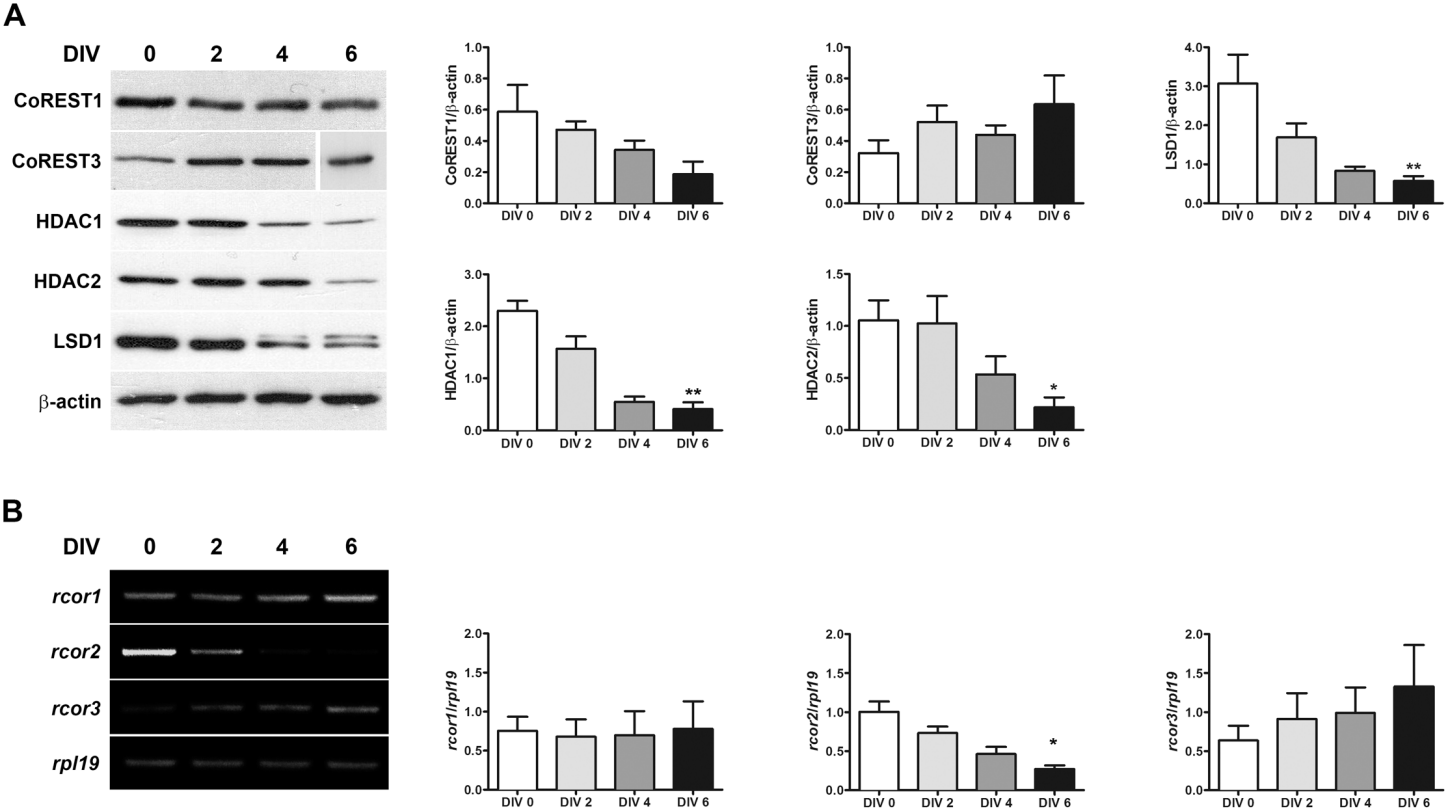
CoREST1 and CoREST2, but not CoREST3 decrease during cortical neurons maturation. (A) Rat embryonic cortical neurons were immediately processed (day 0) or mantained *in vitro* during 2, 4 and 6 days to detect CoREST1, CoREST3, HDAC1, HDAC2 and LSD1. Values correspond to the mean ± SEM. **p<0.01, *p<0.05 (DIV 0 v/s DIV 6). Statistical analysis performed by Kruskal-Wallis test followed by Dunn’s multiple comparison test. (B) Rat embryonic cortical neurons were mantained *in vitro* as described above. Total RNA was extracted and semiquantitative RT-PCR was performed for rat *rcor2* and *rcor3* transcripts; *rpl19* was used as reference gene. Values correspond to the mean ± SEM of at least 3 independent experiments. *p<0.05 (DIV 0 v/s DIV 6). Statistical analysis performed by Kruskal-Wallis test followed by Dunn’s multiple comparison test.

The *in vitro* culture of embryonic cortical neurons during several days is a suitable model of neuronal maturation. In this model, we assessed the expression of members of the LCH complexes. As shown in Fig 4A, CoREST1, LSD1, HDAC1 and HDAC2 protein levels decreased gradually as culture days passed. Similarly to that observed in NGF-induced PC12 cells neuronal differentiation, a larger LSD1 isoform appeared in mature, cultured neurons. On other hand, CoREST3 protein levels were not modified during cortical neuronal maturation, which relates well to that observed at mRNA level in NGF treated PC12 cells. These effects are not caused by glial proliferation as shown by the proportion of GFAP positive cells, which decreases from 3.5% on Div1 to 0% on Div6 in cortical cultures in the presence of AraC (S3 Fig).

To learn about CoRESTs regulation during cortical maturation, we studied *rcor1*, *rcor2* and *rcor3* mRNA expression. In this model, like in NGF-induced PC12 neuronal differentiation, *rcor*2 gene expression decreased gradually in cortical neurons with increasing days in culture. The existence of the rat *rcor1* gene transcript, identified *in silico*, was probed by RT-PCR with specific primers (S4 Fig). RT-PCR showed that *rcor1* mRNA expression remains similar during cortical neurons maturation, suggesting that CoREST1 decrease is due to post-translational modifications rather than transcriptional control. As expected, *rcor*3 mRNA did not change significantly during cortical neurons maturation (Fig 4B).

### Decreased rcor2 gene expression in the mature brain

**Fig 5:**
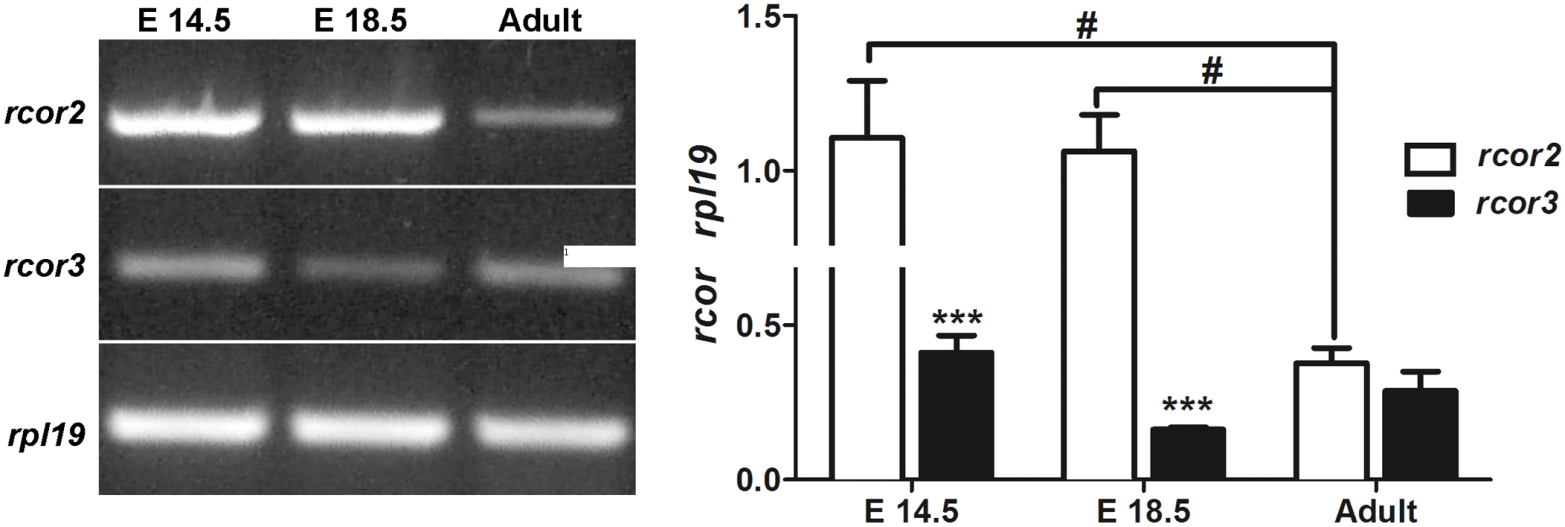
*rcor2* but not *rcor3* is down-regulated during brain development. Total RNA of E.14.5 and E.18.5 embryonic rat brain, and the cortex of adult male rats were subjected to semiquantitative RT-PCR to determine *rcor2* and *rcor3* mRNA expression. *rpl19* was used as reference gene. Values correspond to the mean ± SEM of at least 3 independent experiments. ***p< 0.001, **p<0.01, according to two-way ANOVA and Bonferroni’s posthoc test. # P<0.05, according to one-way ANOVA and Bonferroni’s posthoc test.

To further inquire about rcor genes regulation during brain development, we compared the expression of rcor genes in embryonic days E14.5 and E18.5 respect to the adult rat brain. Two-way ANOVA analysis of rcor2 and rcor3 mRNA levels showed a significant effect of gene (F_(2,15)_ = 35.29 p = 0.0001) and development stage (F_(2,10)_ = 6.36; p = 0.0165), indicating that rcor3 is less expressed than rcor2 during the embryonic phase. However, rcor2 expression is significantly lower in the adult rat brain compared to embryo (Fig 5). Altogether our results strongly indicate that the expression of CoREST proteins is differentially regulated during the neuronal differentiation process and brain development.

### Neurons and glia express CoREST1 and CoREST2 in the adult rat brain

**Fig 6:**
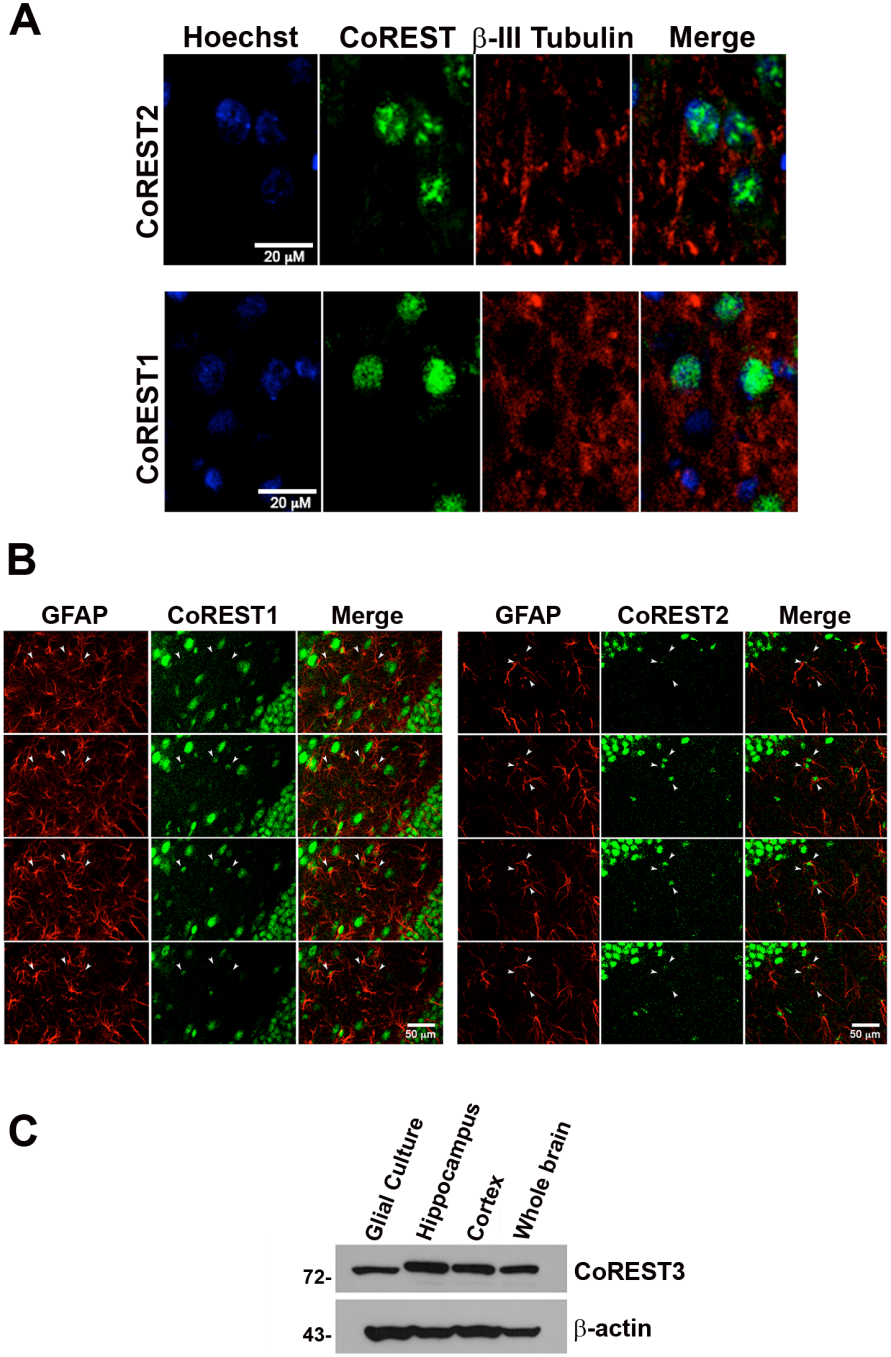
All CoRESTs are expressed in neurons and glia. Adult rat brain sections were incubated with specific antibodies against CoREST1, CoREST2, the neuronal marker β-III tubulin and glial marker GFAP. (A) The immunostaining shows CoREST1 or CoREST2 (green), β-tubulin III (red) and Hoechst (blue, nuclear marker) counterstaining in the Hilar region of the hippocampus. Scale bar = 0 μm. (B) Confocal immunofluorescent images of hippocampus sections from male adult rat brain. The immunostaining shows CoREST1 or CoREST2 (green, as indicated) and GFAP (red). (C) CoREST3 is detected in immunoblots of total protein extracts from mice glial cells in culture and and rat brain areas as indicated.

Previously our group showed that CoREST1, CoREST2, and CoREST3 are expressed ubiquitously in the adult rat brain [8]. To extend this knowledge, we performed colocalization immunofluorescence assays for CoREST1 and CoREST2 with specific markers to distinguish neurons from glial cells. Immunofluorescence assays in sections of adult rat brain showed the presence of cells with positive mark for both CoREST1 and CoREST2 (Fig 6A and 6B). The positive signals are restricted to the cell nuclei and in different nuclei sizes indicating different cell types. Big positive nuclei for CoREST1 and CoREST2 (Fig 6A) that co-express the neuronal specific marker β-III tubulin were observed. Similarly, small nuclei typical of glial cells identified by GFAP expression showed a positive signal for CoREST1 and CoREST2 (Fig 6B). In addition, CoREST3, that was detected by immunoblot with a specific antibody [8] was present in total extracts of cultured glial cells (Fig 6C). The results demonstrate that CoREST1, CoREST2 and CoREST3 are expressed in neuronal and glial cells, suggesting that CoREST proteins exert their functions in both brain cell types.

### CoREST2 and CoREST3 interact with all LSD1 variants including the exclusively neuronal exon 8a-containing LSD1

**Fig 7:**
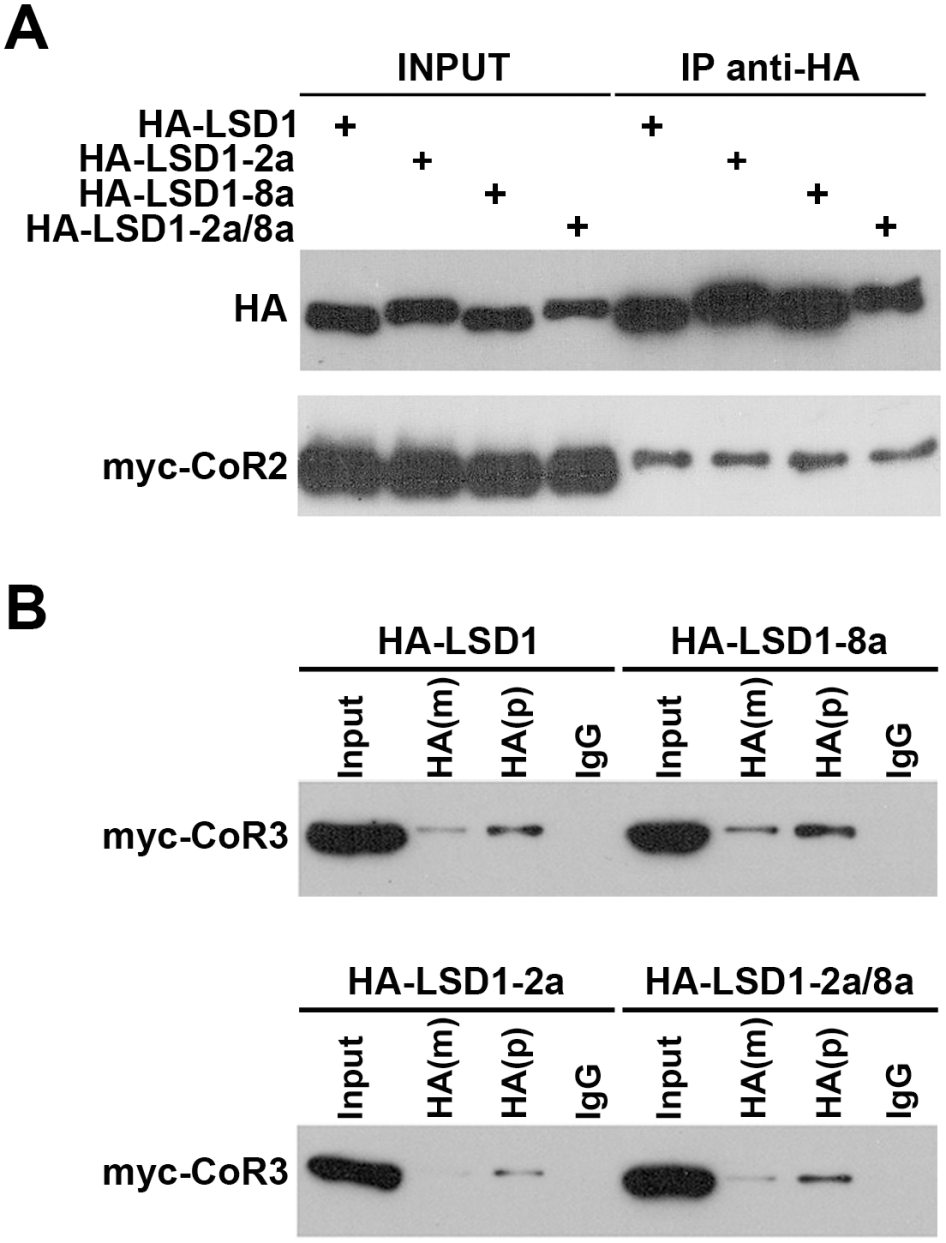
CoREST2 and CoREST3 interact with all LSD1 isoforms. HEK293-T cells were co-transfected with myc-CoREST2 (myc-CoR2) or myc-CoREST3 (myc-CoR3) and HA-LSD1 isoforms (LSD1; LSD1-2a; LSD1-8a; LSD1 2a/8a). Forty-eight hours post-transfection, cells were harvested and total protein extract was immunoprecipitated with the indicated HA antibodies (m: monoclonal; p: policlonal) or IgG (control). Immunoblots were performed with anti-myc or anti-HA antibodies, as indicated.

LSD1 variants containing exon 8a that are expressed exclusively in neurons play relevant roles in neuronal maturation and plasticity processes [3,33,34]. Since, LSD1 demethylase activity depends on its association with CoREST [6,7], co-immunoprecipitation assays were performed to test whether CoREST2 and CoREST3 interact with LSD1 variants, as shown for CoREST1 [3]. The results show that CoREST2 and CoREST3 form complexes with all LSD1 variants (Fig 7A and 7B), suggesting a higher versatility of LCH complexes composition.

## Discussion

The LCH complexes can be formed by several variants of their core components changing their capacity for transcriptional repression [8]. Consequently, shifting the prevalence of some of its components is indicative of functional changes. Here we show that most of the core components of LCH complexes are down-regulated during neuronal differentiation. However, the regulation for each member of the CoREST family is different during this process. A robust reduction of CoREST1, HDAC1 and LSD1 protein levels was observed in PC12 cells differentiated to neurons and *in vitro* maturated cortical neurons. In addition, CoREST2 mRNA decreases significantly in both models. Interestingly, CoREST3 mRNA expression and protein levels do not change during neuronal maturation.

Cell differentiation requires changes in the expression of particular sets of genes; silencing genes that maintain the undifferentiated phenotype, and activating the expression of genes associated to differentiation. These concomitant processes depend on chromatin epigenetic modifications, function performed by complexes that regulate transcription. The LCH complexes may help to maintain undifferentiated phenotypes in precursor cells by repressing the expression of genes of the differentiated phenotype. In this regard, recent work showed that LSD1 and CoREST suppress the differentiation of follicle cell progenitors in flies, until they have completed an appropriate number of divisions [35]. Thus, it is tempting to suggest that reducing specific components of LCH complexes during neuronal maturation is required for the differentiation process.

Previous work has shown that core components of LCH complexes play a significant role in the neuronal differentiation process. Several years ago, it was shown that CoREST1 represses neuronal-specific genes, in interaction with the transcription factor REST/NRSF in neuronal stem cells as well as in non-neuronal cells [36]. Initial reports showed that CoREST1 levels do not change during the differentiation from embryonic stem cells to neurons [12]. However, in agreement with our data, it was shown that CoREST1 suffers an important reduction in the postnatal and adult state compared to embryonic expression in neural territories [14,37]. Recent evidence showed that CoREST1 down-regulation is a necessary event in the maturation and compromise of cortical progenitors, in a mechanism dependent on the regulation of CoREST1 expression by miRNAs [38]. Thus, the reduction of CoREST1 seems to be a regulatory event important for the neuronal differentiation process. Less is known about the role of CoREST2 and CoREST3 in neuronal differentiation. One study showed that *Xenopus laevis rcor2* orthologous (XRCOR2) is expressed predominantly in the embryo neuronal territories. In this work it was also shown that XRCOR2 is able to interact with REST/NRSF, suggesting a redundant role with CoREST1 regulating neuron-specific gene expression. In addition, XRCOR2 through the interaction with the transcription factor ZMYND8 may also regulate neuronal differentiation [15]. In agreement with our data, it was shown that CoREST2 is developmentally regulated in mouse brain, displaying high expression in neural territories in the early embryo, but it reduces towards birth, coincident with the progression of central nervous system maturation [14].

It is intriguing that both CoREST1 and CoREST2 are down-regulated, while CoREST3 remains low expressed but unchanged during neuronal differentiation. We have shown that the LCH complexes formed with different members of CoREST family exhibit different repressor capacities. Complexes formed by CoREST2 and CoREST3 have significant lower transcriptional repression capacity compared with LCH complexes formed by CoREST1 [4]. It is possible that CoREST1 and CoREST2 containing complexes play different functional roles during neuronal differentiation. Our finding that CoREST2 and CoREST3 are interacting partners of all LSD1 isoforms increases the range of regulatory possibilities of LCH complexes. Interestingly, it was shown that the shortest splicing variant of CoREST3, which lacks SANT2 domain, displays a dominant-negative role counteracting CoREST1 repressive function during blood cells differentiation [9]. It is noteworthy that although the reduction in protein components of the LCH complex is evident, they are not completely eliminated. In fact, our present and previous work shows that all CoRESTs are expressed in the mature brain. Here, we extended the information, showing that all CoRESTs are expressed in neurons and glial cells. In addition, the analysis of databases shows that all CoRESTs are detectable in brain, including the four splicing variants of CoREST3.

Our results also show that LSD1 and HDAC1/2 decrease during neuronal differentiation, confirming other reports. Recent evidence suggests a dual role for HDAC function in different phases of human neuronal stem cells phenotypic commitment. HDAC activity inhibition is required to maintain pluripotency during DIV 0–4; conversely neuronal differentiation is potentiated by HDAC inhibition during DIV 4–8 [39]. Accordingly, the strong decrease of HDAC1/2 protein levels in differentiated PC12 cells and *in vitro* cortical neurons maturation, strongly suggest that final steps of neuronal phenotype acquisition requires a decrease of HDAC-dependent transcriptional repression. Our data also show a significant decrease of the total amount of LSD1 protein levels in differentiated PC12 cells and mature cortical neurons. In addition, our data showing the appearance of a higher molecular weight band suggests that either variants of LSD1 containing the 2a exon or a modified isoform of LSD1 may also have a higher prevalence in mature neurons. Recent evidence demonstrates that LSD1 is required for neuronal progenitor cells maintenance during cortical development by specific repression of Atrophin 1 gene expression [40]. In this study, LSD1 knock down drove to neuronal progenitors to premature differentiation, supporting the idea that LCH complexes decrease is a required process for neuronal differentiation.

In conclusion, neuronal differentiation is accompanied by decreased expression of all core components of LCH complexes, but not CoREST3. The changes in the proportion of core components forming the LCH complexes, added to their different transcriptional repression capacity suggest important and distinct roles of these complexes in neural progenitors and mature brain.

## Supporting Information

S1 Fig CoREST3 protein domains at human and mouse *rcor3* transcripts. The UCSC Genome Browser images shows the genomic positions were CoREST3 protein domains are encoded at human and mouse genomes (A and B, respectively).
(TIF)

S2 Fig NGF-induced neuronal differentiation of PC12 cells and maturation of embryonic cortical neurons *in vitro*. (A) PC12 cells treated with NGF (50 ng/ml) for 7 days and embryonic cortical neurons (E.18.5) maintained *in vitro* during 6 days (top and Bottom, respectively). (B) Immunoblot for the neuronal differentiation marker GAP43 from protein extracts of PC12 cells treated with NGF (50 ng/ml) for 7 days. Quantification of one experiment made in triplicate.
(TIF)

S3 Fig Absence of glial cells proliferation in primary culture of cortical neurons *in vitro*. (A) Immunofluorescence assays from primary culture of cortical neurons using anti- ß-III Tubulin (neuronal marker) and GFAP (glial marker) in presence or absence of AraC in the culture medium. (B) Quantification of positives cells for each specific marker, showing the mean ± SEM percentage of positives cells from at least 4 different fields in each day *in vitro*.
(TIF)

S4 Fig RT-PCR amplicons location at *rcor1-3* transcripts. The location of each amplicon is indicated by intervals where vertical black lines represent the primer position and the arrow heads represent the amplicon direction.
(TIF)
