# Correction: Decreased Expression of CoREST1 and CoREST2 Together with LSD1 and HDAC1/2 during Neuronal Differentiation

**DOI:** 10.1371/journal.pone.0133555

**Published:** 2015-07-17

**Authors:** The PLOS ONE Staff

There is an error in Fig 5. A white strip appears in the center of the figure. The publisher apologizes for the error. Please view the correct figure here.

**Fig 5:**
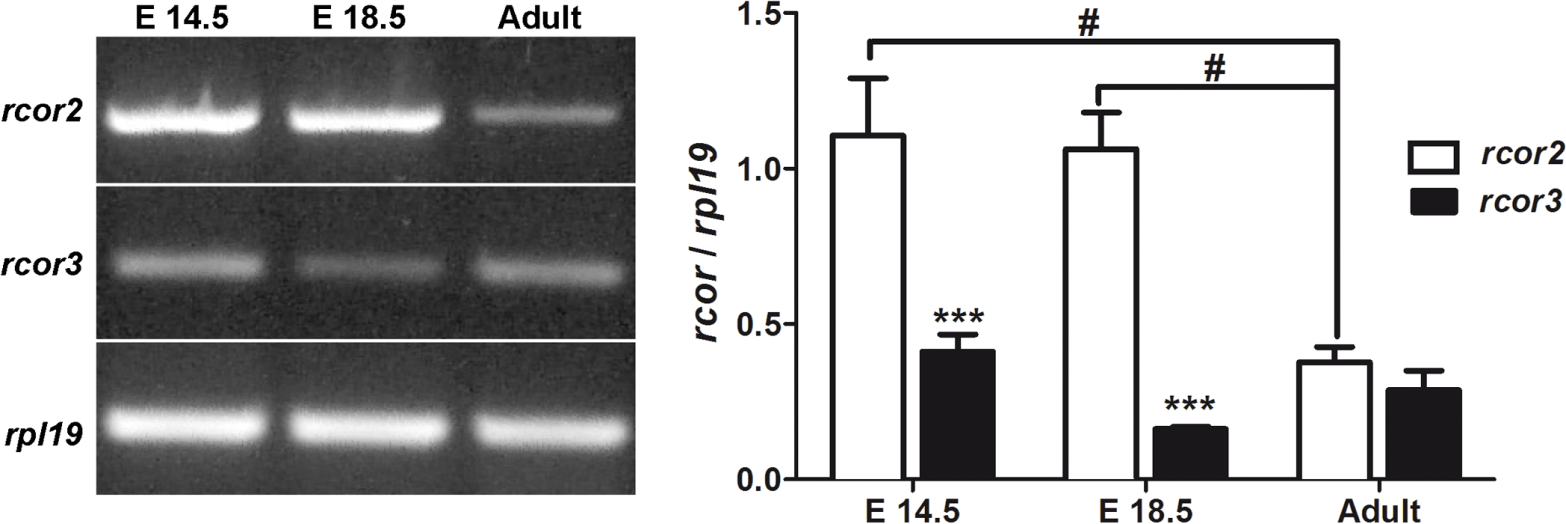
*rcor2* but not *rcor3* is down-regulated during brain development. Total RNA of E.14.5 and E.18.5 embryonic rat brain, and the cortex of adult male rats were subjected to semiquantitative RT-PCR to determine *rcor2* and *rcor3* mRNA expression. *rpl19* was used as reference gene. Values correspond to the mean ± SEM of at least 3 independent experiments. ***p< 0.001, **p<0.01, according to two-way ANOVA and Bonferroni’s posthoc test. # P<0.05, according to one-way ANOVA and Bonferroni’s posthoc test.
