# Supplementary figures and images for: Decreased Expression of CoREST1 and CoREST2 Together with LSD1 and HDAC1/2 during Neuronal Differentiation
Source: PLoS One. 2015 Jun 25;10(6):e0131760. doi: 10.1371/journal.pone.0131760 (PMC4482511; doi:10.1371/journal.pone.0131760)

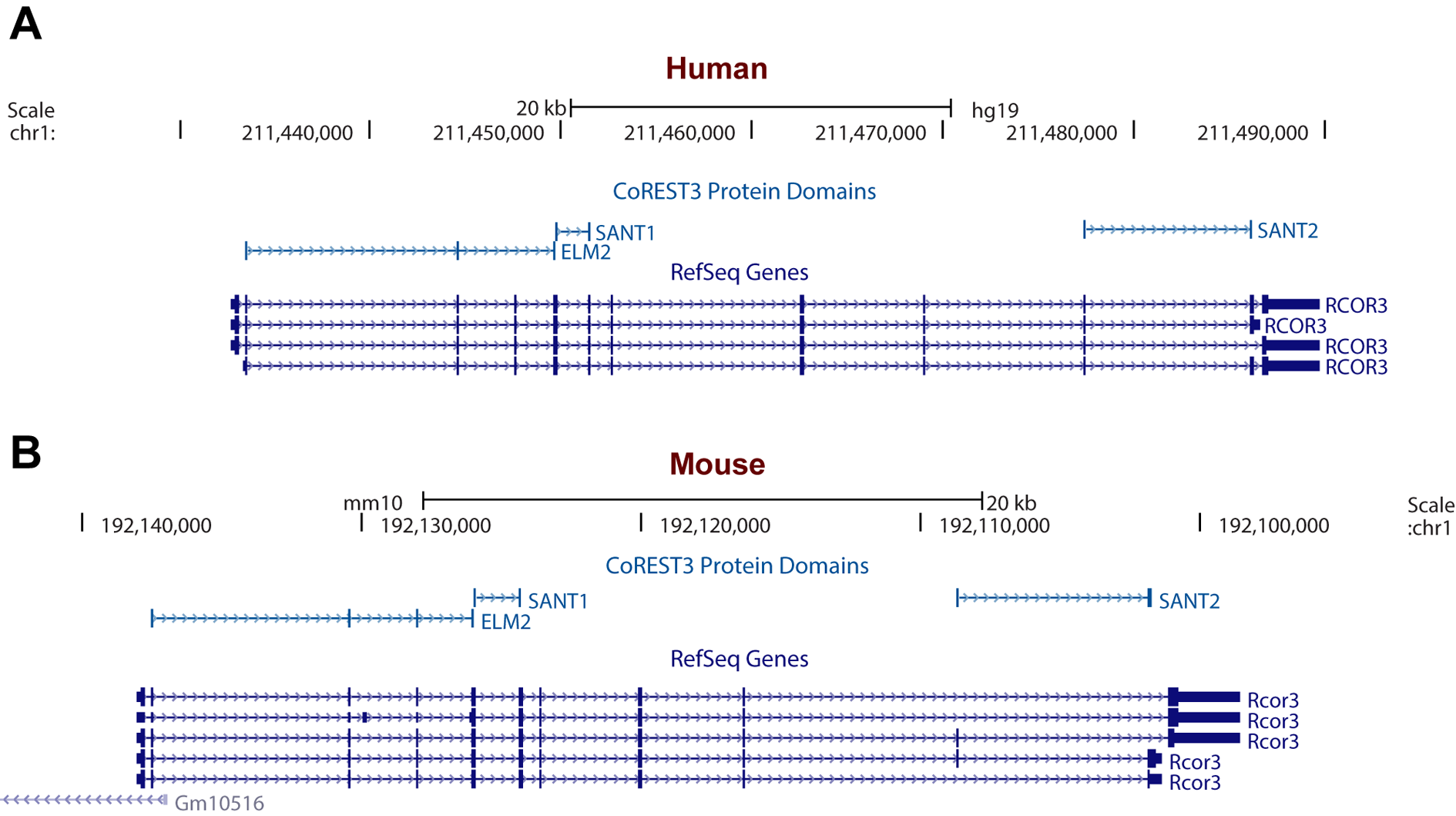

Supplement: S1 Fig — The UCSC Genome Browser images shows the genomic positions were CoREST3 protein domains are encoded at human and mouse genomes (A and B, respectively). (TIF) [file pone.0131760.s001.tif]

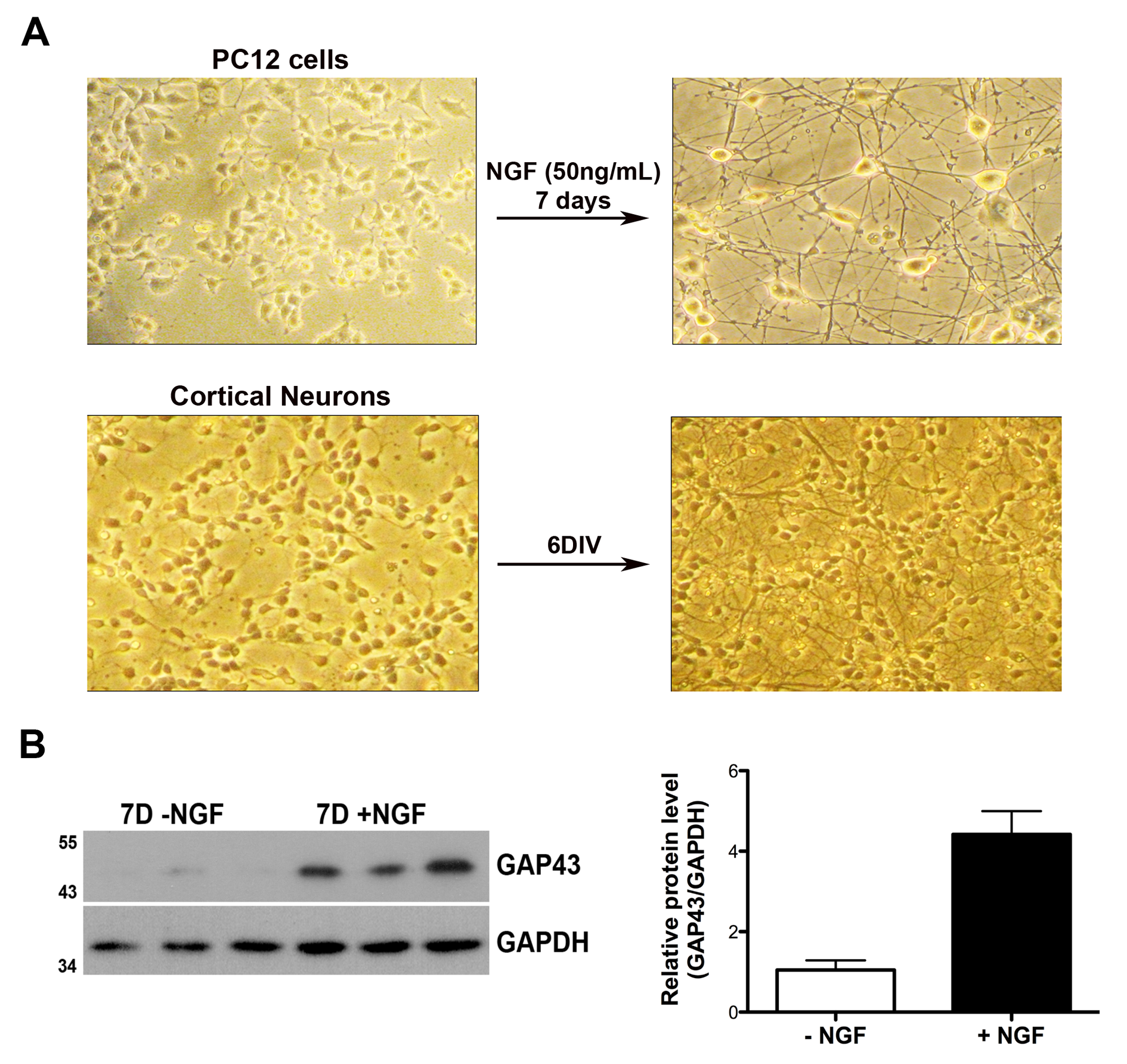

Supplement: S2 Fig — (A) PC12 cells treated with NGF (50 ng/ml) for 7 days and embryonic cortical neurons (E.18.5) maintained in vitro during 6 days (top and Bottom, respectively). (B) Immunoblot for the neuronal differentiation marker GAP43 from protein extracts of PC12 cells treated with NGF (50 ng/ml) for 7 days. Quantification of one experiment made in triplicate. (TIF) [file pone.0131760.s002.tif]

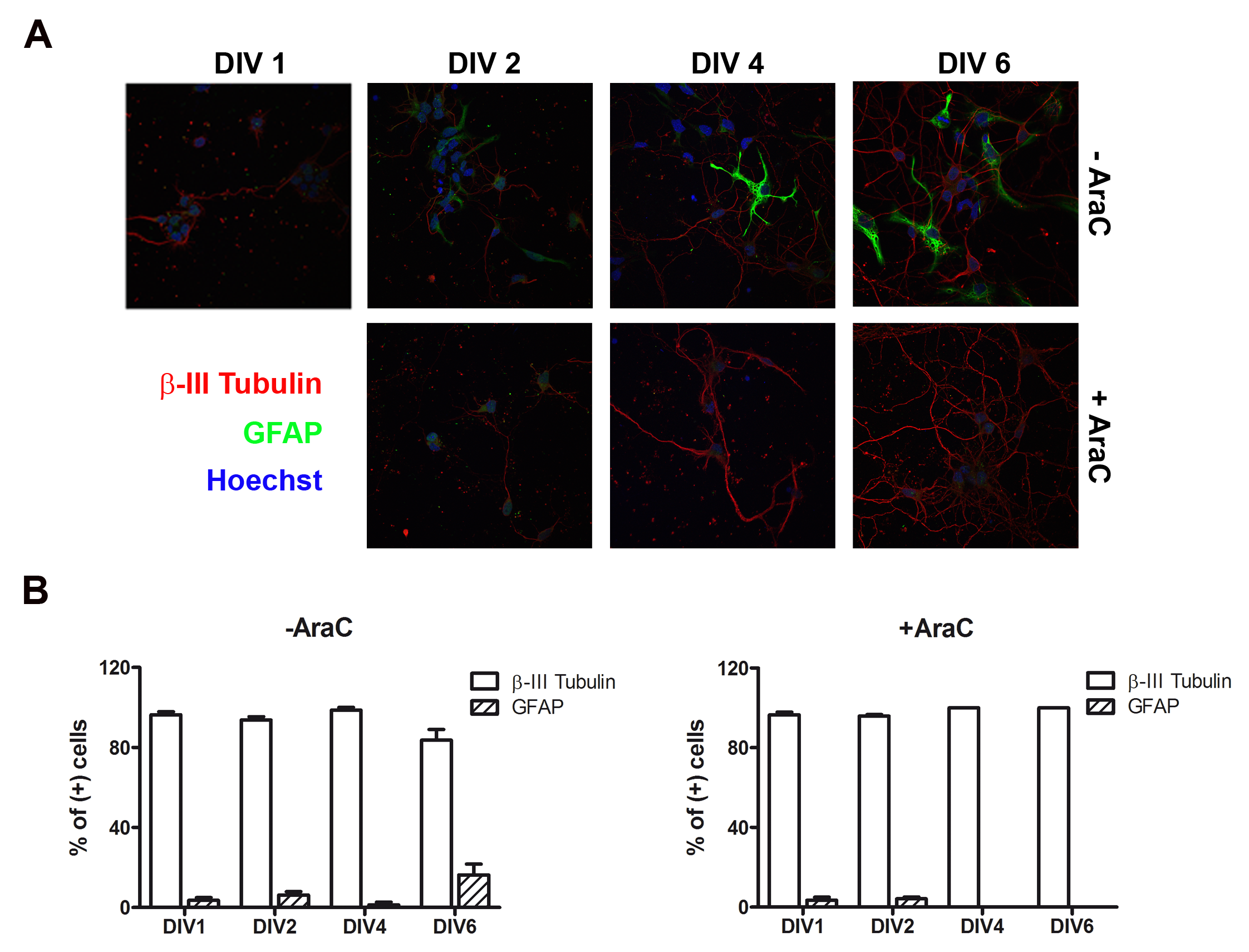

Supplement: S3 Fig — (A) Immunofluorescence assays from primary culture of cortical neurons using anti- ß-III Tubulin (neuronal marker) and GFAP (glial marker) in presence or absence of AraC in the culture medium. (B) Quantification of positives cells for each specific marker, showing the mean ± SEM percentage of positives cells from at least 4 different fields in each day in vitro. (TIF) [file pone.0131760.s003.tif]

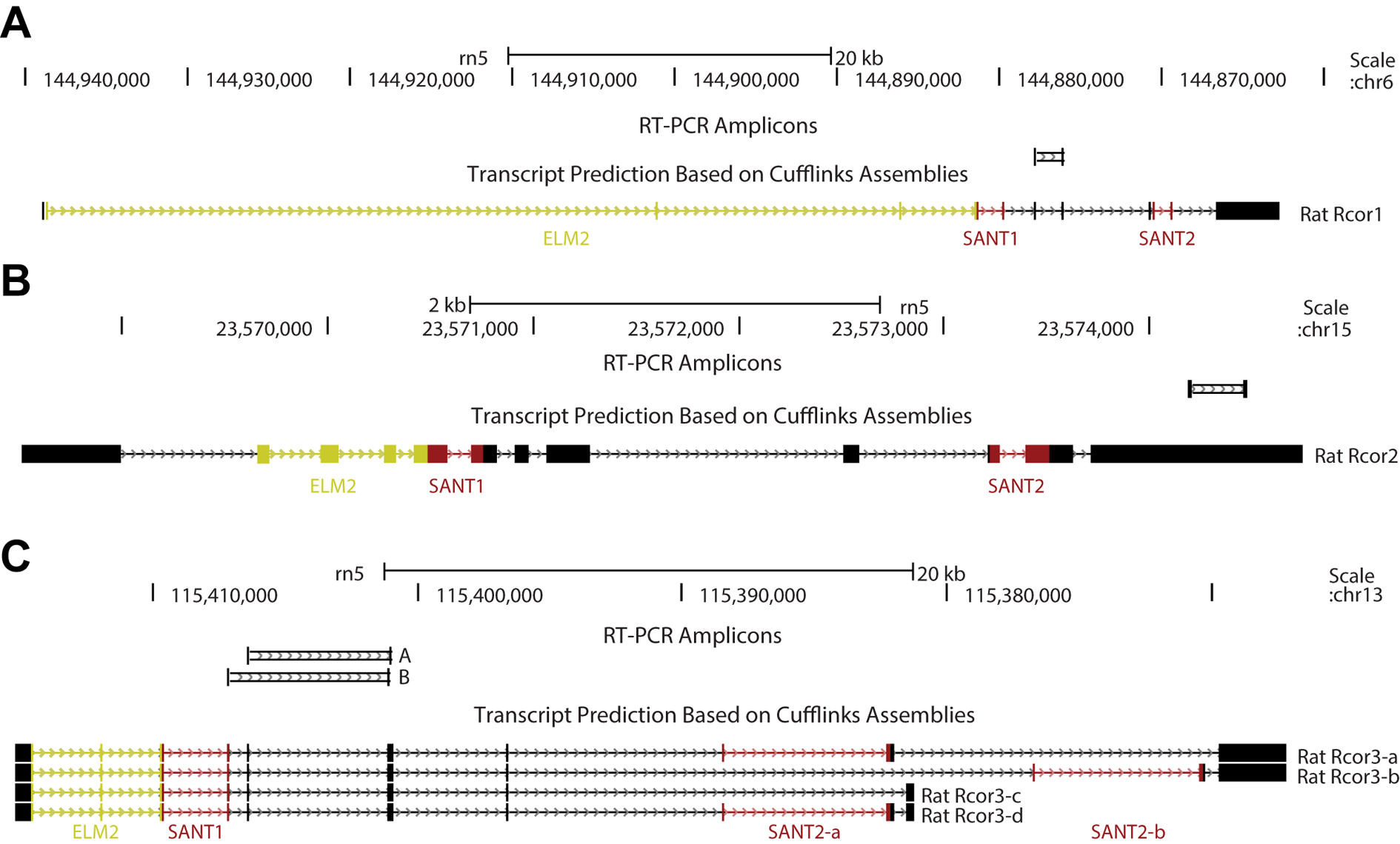

Supplement: S4 Fig — The location of each amplicon is indicated by intervals where vertical black lines represent the primer position and the arrow heads represent the amplicon direction. (TIF) [file pone.0131760.s004.tif]
